# Physiologically informed neuromodulation

**DOI:** 10.1016/j.jns.2021.120121

**Published:** 2022-03-15

**Authors:** Karen Wendt, Timothy Denison, Gaynor Foster, Lothar Krinke, Alix Thomson, Saydra Wilson, Alik S. Widge

**Affiliations:** a Department of Engineering Science and MRC Brain Network Dynamics Unit, University of Oxford, Oxford, UK; b Welcony Inc., Plymouth, MN, United States of America; c Department of Neuroscience, School of Medicine, West Virginia University, Morgantown, WV, United States of America; d Department of Psychiatry and Behavioral Sciences, University of Minnesota-Twin Cities, Minneapolis, MN, United States of America; e Medical Discovery Team on Additions, University of Minnesota, Minneapolis, MN, United States of America

**Keywords:** Neuromodulation, Biomarkers in psychiatry, Brain-state dependent stimulation, Deep brain stimulation, Transcranial magnetic stimulation, Electroencephalography

## Abstract

The rapid evolution of neuromodulation techniques includes an increasing amount of research into stimulation paradigms that are guided by patients' neurophysiology, to increase efficacy and responder rates. Treatment personalisation and target engagement have shown to be effective in fields such as Parkinson's disease, and closed-loop paradigms have been successfully implemented in cardiac defibrillators. Promising avenues are being explored for physiologically informed neuromodulation in psychiatry. Matching the stimulation frequency to individual brain rhythms has shown some promise in transcranial magnetic stimulation (TMS). Matching the phase of those rhythms may further enhance neuroplasticity, for instance when combining TMS with electroencephalographic (EEG) recordings. Resting-state EEG and event-related potentials may be useful to demonstrate connectivity between stimulation sites and connected areas. These techniques are available today to the psychiatrist to diagnose underlying sleep disorders, epilepsy, or lesions as contributing factors to the cause of depression. These technologies may also be useful in assessing the patient's brain network status prior to deciding on treatment options. Ongoing research using invasive recordings may allow for future identification of mood biomarkers and network structure. A core limitation is that biomarker research may currently be limited by the internal heterogeneity of psychiatric disorders according to the current DSM-based classifications. New approaches are being developed and may soon be validated. Finally, care must be taken when incorporating closed-loop capabilities into neuromodulation systems, by ensuring the safe operation of the system and understanding the physiological dynamics. Neurophysiological tools are rapidly evolving and will likely define the next generation of neuromodulation therapies.

## Highlights

• Physiologically informed neuromodulation is promising to improve therapy efficacy.
• Examples of effective approaches exist for several neurological diseases.
• Research into implementations for psychiatric applications is advancing rapidly.
• Further research and validation are needed before using these in clinical practice.

## 1 The need for physiologically informed neuromodulation

The field of neuromodulation therapies and technologies is expanding rapidly. Neuromodulation devices, especially neuromodulation devices that deliver electrical current to neuronal tissue, can deliver a myriad of possible stimulation parameters. In addition, these technologies can target specific anatomic targets. This offers the prospect to personalize therapy to a patient's individual needs. Neurophysiological biomarkers such as electroencephalographical signals (EEG) or local field potentials (LFPs) offer the prospect to better personalize or even automate the selection of stimulation parameters or physiological stimulation targets.

For example, an implantable Responsive Neurostimulation System (RNS) that utilizes electrophysiological signals to trigger the stimulation had been cleared by the Food and Drug Administration (FDA) in 2013 for the treatment of epilepsy [1]. A vagal nerve stimulation system triggered by ictal tachycardia is also available to patients with epilepsy [2]. A deep brain stimulation system that measures local field potentials has been used in several clinical trials and has been cleared by the FDA in 2020 [3]. Spinal cord stimulation systems are emerging that measure a patient's posture or compound action potentials to control stimulation intensity.

Medical devices that automatically adjust therapy are currently available in other fields. For example, an implantable cardio defibrillator uses several sensors to ensure that a patient receives defibrillation only when required. Similarly, wearable insulin pumps that are controlled by glucose sensors have greatly improved the lives of diabetes patients.

As of today, there are no FDA-cleared biomarkers that aid in the selection of stimulation parameters or stimulation targets in psychiatric neurostimulation therapies, whether non-invasive such as repetitive transcranial magnetic stimulation (rTMS) or invasive such as deep brain stimulation (DBS). Such devices are already approved (TMS for depression and obsessive compulsive disorder (OCD), DBS for OCD) and many new trials are ongoing, meaning that biomarkers to improve treatment precision are increasingly necessary.

Research is ongoing to identify biomarkers that could be used for neuromodulation therapies. The therapeutic goals include:

1) Patient stratification: Prediction of which patients are likely responders to neuromodulation, or of which form of neuromodulation is right for a patient
2) Stimulation target identification: customization of a neuromodulation therapy target (anatomically or physiologically defined) to a patient's individual disease state or brain state
3) Stimulation parameter personalization: Biomarkers that inform which stimulation parameters lead to therapy success. These parameters include stimulation amplitude, pulse width and shape as well as stimulation frequency and stimulation patterns. Some biomarkers might provide insight within seconds while others will change only over longer time periods.

EEG is currently used in some psychiatric cases to rule out underlying neurological conditions such as epilepsy, tumors or other neurological disorders. Similarly, EEG-based sleep staging can be used to diagnose and treat sleep disorders that interact with mood state, e.g. identifying obstructive sleep apnea that is causing treatment-resistant depression or fatigue. EEG based biofeedback is often used in patients with attention deficit disorders [4], although the evidence quality remains low [5,6]. There is also great interest in further development, e.g. biomarkers for Autism Spectrum Disorder have been accepted by the FDA biomarker program for evaluation as possibly valid clinical trial metrics.

Many tools are available today to the psychiatrist, and the technology and clinical evidence is developing quickly. These tools may provide value as the psychiatrist is evaluating the overall brain health state of a patient. It is important for clinicians to familiarize themselves with these technologies as rapid progress is expected over the next few years.

## 2 Potential roles of neurophysiologic biomarkers

### 2.1 Biotyping/endophenotypes/prediction

Given the biologic heterogeneity of mental illness, there has been great hope that neurophysiology could directly classify patients into “biotypes” (endophenotypes) whose underlying physiology is more homogenous, and thus who might show a more uniform response to neuromodulation [[7], [8], [9]]. Even before the rise of neuromodulation as a clinical treatment, EEG was used as a source of phenotypic and predictive markers [7,10]. Early attempts were limited by small sample sizes [7], but large multi-center studies have now created databanks that may enable discovery [11,12]. The majority of this work has targeted medication response, but newer papers also attempt to predict response to TMS [7,9,13]. Work with more invasive methodologies, such as DBS, has focused more on proving target engagement (see below).

On the surface, the biotyping/prediction literature is quite positive, with many studies reporting reliable discrimination of responders from non-responders [7,10]. More recent work, however, suggests that simple physiologic markers may have limited value in treatment selection. There is a substantial publication bias, and most published studies have very small samples [7]. This likely means that most published markers represent effect size inflation or analytic coincidence. Large datasets have enabled more sophisticated marker/biotype discovery, largely based on analysis of connectivity patterns across many sensors/brain regions [9,13,14]. It remains unclear whether these patterns will be replicable. They can be unstable across re-analyses within the same dataset [15], and the within-individual test-retest reliability of network markers is often much worse than simpler, power- based metrics [[16], [17], [18]]. In a recent set of fMRI studies, TMS-predictive biotypes were suggested to be mainly an artifact of analytic technique [19]. The internal heterogeneity of psychiatric disorders may make it largely impossible to identify reliable prognostic markers for any categorical DSM-based diagnosis [20,21]. Alternate classification systems have been proposed that may be more reliable [[21], [22], [23], [24]], but these are many years away from being clinically available. Until then, the value of physiology in neuromodulation may lie in fine-tuning the treatment dose, rather than in patient selection.

### 2.2 Treatment personalization

Psychiatric neuromodulation emphasizes a relatively narrow, one-size-fits-all set of parameters. Clinical TMS is delivered almost exclusively in 10 Hz or theta-burst stimulation (TBS) paradigms [25,26], DBS almost exclusively at about 130 Hz [[27], [28], [29], [30]], and vagal nerve stimulation (VNS) at 20 or 30 Hz [31]. Individual patients, however, show variation in the peak frequencies of endogenous brain rhythms, a “brain fingerprint” that remains stable over time [32,33]. There is theoretical and empirical evidence that neuromodulation interacts with these rhythms, in a frequency-dependent manner [34,35]. Thus, treatment outcomes might be improved by taking these endogenous frequency variations into account.

For instance, the individual alpha frequency (IAF) is an EEG measurement commonly recorded in psychiatric research. Tuning rTMS to this frequency has been hypothesized to increase its effectiveness [36]. This has been demonstrated in schizophrenia patients, where stimulation at the IAF produced a greater therapeutic effect on negative symptoms [37] and on both positive symptoms and depressive symptoms [38] than sham or other set frequencies. In depression, a correlation between the IAF and the response to rTMS has been suggested in some studies [39] but failed to be replicated in others [40]. Another study in MDD patients showed that the proximity of the IAF to the stimulation frequency of 10 Hz, rather than the value of the IAF itself, was associated with a better treatment response after rTMS applied to the left DLPFC [41]. This was one of the only psychiatric biomarkers to survive an independent replication [42].

These findings in rTMS led to the development of synchronized transcranial magnetic stimulation (sTMS), a non-depolarizing form of TMS. sTMS creates a sinusoidally changing magnetic field, generated by rotating a set of magnets. The rotational speed is set so that the magnetic field's frequency matches each patient's IAF. Initial reports of sTMS compared MDD clinical outcomes in patients treated with a rotational speed tuned to the subjects personal average IAF to that of subjects treated with a rotational speed at a random frequency in the alpha range (8–13 Hz). The random protocol was as clinically effective as the IAF personalized speed [43]. A second group performed a larger trial of sTMS, which did not show separation from sham [44]. Post hoc analysis, however, found that patients with IAF in the highest quartile, 10.46–12.71 Hz, had significantly greater clinical improvement than patients with IAF in the lowest quartile, 8.0–9.08 Hz. Clinical improvement was greater in each quartile as the personalized stimulation frequency became closer to ≥10 Hz [45]. It remains unclear why synchronization near 10 Hz is more impactful than synchronization at other frequencies.

Animal studies also support the concept of matching stimulation to endogenous frequencies or resonances. Coordinated reset (CR) is a novel approach to DBS for Parkinson's disease that attempts to de-synchronize pathological oscillatory generators in the motor circuitry [46,47]. In monkey studies, relatively brief applications of CR sequences produced long-lasting motor improvement, in comparison to traditional DBS effects that disappeared when stimulation stopped [48]. Pathologically strong oscillations are also argued to be present in psychiatric disorders [[49], [50], [51], [52]], and similar techniques might effectively disrupt them. For other disorders marked by overly weak oscillations (e.g., hypothesized fronto-amygdalar disconnects in trauma and anxiety disorders), there are techniques to boost cross-regional oscillatory synchrony by resonance-informed stimulation customized to individual brains [53]. Mathematical approaches are just emerging to automatically and optimally compute the stimulation parameters that can best engage an individual patient's oscillations [54,55]. As these mature, they may dramatically alter clinical neuromodulation practice.

### 2.3 Target engagement

Target engagement in neuromodulation is confirmed when three essential elements have been verified: 1)The stimulus reaches the target location, 2) the stimulus modulates the activity of the target region through changes in an objective biomarker (preferably in a dose-dependent fashion), and 3) the biomarker changes track clinically relevant/subject measures. Target engagement is valuable because, if confirmed, it elucidates underlying mechanisms of action that enable more efficient neuromodulation protocols. Demonstrating the three elements of target engagement can require the use of multiple modalities. For example, electric field modeling can demonstrate the depth and focality of a neuromodulation technique [56]. Functional imaging studies and electrophysiological measures have generated an array of putative biomarkers, as noted above [57]. Target engagement metrics are critically important for psychiatric neuromodulation because the clinical response is delayed, on the order of weeks to months after stimulation onset. This limits the pace of treatment adjustment, and may mean that some patients never achieve an adequate “dose” of the intervention [7,21,26]. If we can optimize target engagement via a rapidly measurable physiological signal, clinical outcomes may improve. Proof of target engagement will also become more necessary with the rise of very intensive protocols such as accelerated rTMS using multiple treatment sessions per day [58,59]. The high financial and time burdens of these protocols will require higher assurance of successful dosing.

The most straightforward metric of target engagement is the evoked or event-related potential (ERP), which is a stereotypical, reproducible electrical response to a specific stimulus. ERPs are particularly valuable in neuromodulation because they act as measures of stimulation intensity relative to tissue/circuit excitability. The motor response commonly used to determine rTMS dosing is, effectively, an ERP read out indirectly. When neuromodulation is combined with EEG measurement, ERPs can be used to directly measure cortical responses and reactivity. For instance, paired pulse facilitation of motor cortex excitability has been proposed as a marker of TMS responsiveness. Two recent small studies in MDD provided preliminary evidence that greater modulation of motor cortex excitability prior to rTMS predicted a better antidepressant response [60,61]. Similarly, amplitude of motor evoked potentials (MEPS) prior to TMS for Alzheimer's disease predicted clinical outcome even when controlling for brain atrophy at the motor area [62]. These results are preliminary, but exciting if replicated, because motor cortex excitability measures are simple to perform using equipment already available in many clinical TMS offices.

In invasive neuromodulation, ERPs can be used to map connectivity between stimulation sites and connected areas [63]. This can be particularly useful in invasive neuromodulation, where there is a growing understanding that clinical effects require network engagement [21,64]. ERPs complement other forms of connectivity estimation such as tractographic imaging [65], and because they can be quickly measured in clinic, can easily form the basis of a real-time stimulator programming algorithm. In DBS for Parkinson's disease, EEG-based ERP mapping can demonstrate cortical engagement that correlates with both response [66] and side effects [67]. Similar techniques are being explored in DBS for depression [68], with evidence of test-retest reliability. ERPs provoked by disease-relevant stimuli could also be used as a readout of target engagement, e.g. showing changes in emotional processing from stimulation meant to treat depression. Hajcak and colleagues demonstrated this during lateral prefrontal cortex epidural cortical stimulation (EpCS) in depression, showing suppression of an ERP related to aversive visual stimuli [69].

Brain oscillations, noted above as a means for treatment personalization, might also show target engagement. The strongest evidence of this is in PD, using basal ganglia beta activity (13–30 Hz). The amplitude of the beta oscillations correlates with the level of motor impairment and its reduction through DBS correlates with the improvement of motor symptoms [70,71]. Adaptive DBS systems, which only stimulate when the power in the beta frequency band oscillations exceeds a threshold [72] or that adjust the stimulation level proportionally to the amplitude of the oscillations [73,74], have been shown to improve motor symptoms while reducing energy usage and side effects compared to continuous DBS [72,75]. There is a long literature of attempts to find similar biomarkers for TMS [10,57], but these have not replicated well [7,40]. Next-generation invasive devices have permitted direct recording of brain oscillations from subcortical structures [52,76,77], but the sample sizes remain too small to identify robust markers.

Newer approaches, which are complex but promising, apply large-scale invasive recordings to identify psychiatric biomarkers. One recent paper suggested that a weighted mixture of power-band signals from multiple limbic regions could predict mood fluctuations over hours to days [78]. This approach and others like it are in active clinical trials [79,80]. One major limitation is that the reported markers require simultaneous recordings from tens to hundreds of channels, which cannot be achieved with current neuromodulation devices. That might be overcome by focusing on more narrow symptom definitions rather than ill-defined subjective constructs such as mood. For instance, specific forms of cognitive impairment could be read out from similar power-band changes using as few as five recording channels [81,82].

Finally, physiologic measures may also be used to compute synchronization between two sites, e.g. between a stimulation target and its partners in disease-relevant circuits. To the degree that brain disorders are network disorders, these connectivity metrics may be more clinically relevant read-outs. This is again most advanced in PD, where phase-amplitude coupling between the primary motor cortex subthalamic nucleus β-phase has been proposed as a mechanism for DBS [83]. Attempts to apply the same thought process to psychiatric disorders include cortico-striatal circuit measurements in OCD [52] and an amygdala-hippocampus network whose variability correlates with mood [51]. Although still highly investigational, these synchrony measures are exciting, because as noted above, other groups are developing specific stimulation protocols to change cross-region synchrony. The combination of these lines of research may lead to new forms of network-informed neuromodulation [21,50].

## 3 Closed loop, physiologically informed therapy

**Fig. 1:**
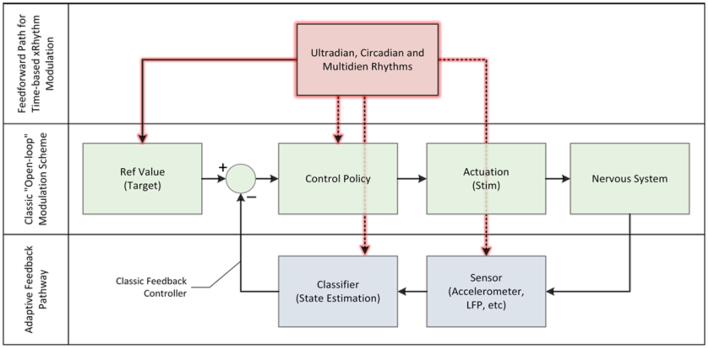
Modeling open-loop, closed-loop and feed-forward control of the neurostimulator. The highlighted arrows capture the future state where devices respond to temporal rhythms, as a digital chronotherapy.

Beyond using physiology to select a single static “dose” for each patient, physiological signals can be used also to adjust stimulation based on the immediate needs of the patients. Referencing Fig. 1, the majority of systems currently run in an “open-loop” mode of operation, meaning that the device provides a stimulation regime that is configured by a clinician. The clinician sets the “control policy,” in control theory terms, to achieve a desired outcome defined as the reference. The patient might have some marginal control (e.g., in an implant, to select among pre-configured patterns, adjust stimulation, or turn the system on and off) but a prescribed pattern is generally static. A first approach to closed-loop is to measure the defined biomarkers, classify them, and then adjust the stimulation according to an algorithmic control policy. This policy reflects the actions that a clinician would prescribe, but in an automated format and with much more frequent adjustment than is possible in clinic. This method is illustrated as the adaptive feedback pathway.

Neuromodulation systems are now incorporating closed-loop, adaptive capabilities [84]. Because some seizures are associated with acceleration in heart rate, a heart rate sensor has been incorporated into vagus nerve stimulation devices so that stimulation is activated when the heart rate exceeds a predetermined threshold. A spinal cord stimulation system uses an embedded three-axis accelerometer to dynamically adjust the pain stimulator's amplitude based on changes in posture and activity. Perhaps the most well-known example is a direct-brain responsive neurostimulator, the RNS, for the treatment of epilepsy [85]. LFP activity is continuously monitored using electrodes placed in the region of seizure onset. Stimulation is provided only when epileptiform activity is detected, reducing the amount of stimulation from hours a day as is the case for open loop devices, to about 3 min total on average per day. These examples illustrate the ability of neuromodulation therapies to adjust stimulation dynamically.

Responsive neuromodulation is also an area of active research and development. Clinical trials are underway to adapt DBS systems to respond to changes in electrophysiological signals. For Parkinson's disease, the model is to titrate stimulation according to aberrant oscillations in the basal ganglia, most notably the beta or gamma rhythms [75,86]. When oscillations exceed a threshold amplitude, stimulation is titrated accordingly to drive them to a more neurotypical state. When there is a reduction in oscillations, for example, when medication is taken, the beta rhythm might naturally drop or gamma increase, and so the stimulation is turned down. A strength of the latest studies is that the algorithm is tested in well-controlled clinical trials; in addition to using open loop controls, a control pattern is stored and then delivered at times that are uncorrelated to physiological signals. The additional control establishes that the responsive algorithm's linkage of stimulation to measured physiology state is critical to operation, as opposed to a clinical benefit arising solely from intermittent stimulation patterns; it is worth noting this control is yet to be applied in brain responsive epilepsy devices. Similar concepts are being explored for depression, OCD, and essential tremor [87,88].

In addition to responding to latent signals in the nervous system, devices can also respond to evoked potentials. The response of the nervous system to stimulation pulses can be detected and used to adjust stimulation on a pulse-per-pulse basis [89]. This optimization approach is being explored for the improvement of spinal cord stimulation, building off the first-generation of systems using accelerometers.

The inherent time variation of physiology is also entering into algorithms. An exciting area to consider is how stimulation might be adapted according to biological rhythms, such as circadian/diurnal rhythms and multi-day rhythms. Most devices today do not modify therapy according to the sleep-wake cycle, even though disease activity may vary between sleep and waking states. Researchers have used long-term intracranial EEG data collected from a brain-responsive neurostimulator to identify periodicities in epileptiform activity and in seizures to understand how to modify treatment according to a patient's individual cycles, and even, perhaps, to forecast times of greater susceptibility to seizures [90,91]. Similar research is underway in movement disorders for modification of diurnal stimulation based on that patient's sleep-wake cycle to enhance both sleep architecture and daytime vigilance. In the future, as shown in Fig. 1, the algorithms in devices will integrate both circadian feedforward adjustments to the stimulation pattern and short-time responsive modes, much like natural control mechanisms work to regulate physiology [92].

Time also enters into the response dynamics that are allowed by a closed-loop system. The current approaches to Parkinson's disease and tremor are possible because motor symptoms are relatively obvious and change rapidly in response to stimulation. On the other hand, epilepsy devices are challenged by the response time that can extend from months to years for effect. What about for psychiatric disorders, where we may need to focus on modifying underlying network problems and faulty circuits, and where subjective symptoms may only change slowly? As a basic rule-of-thumb, we must be cautious not to try and adjust stimulation faster than the response time of the system. Moving too quickly (failing to understand what a control engineer would term the dynamics of a system) could lead to stimulation that is ineffective or actively harmful, even if the same parameters might be helpful when applied with slower rates of change.

## 4 Advanced applications of physiology

On the other hand, an understanding of physiological system dynamics could lead to more effective stimulation parameters. A particularly promising approach is the exploration of timing-dependent plasticity. Brain circuits are constantly changing, and generally, the connections between regions become stronger when those regions are consistently active at the same time [93]. Early studies in primates showed that this principle can be exploited to design physiologically-informed stimulation that induces neuroplasticity. In these studies, closed loop stimulation was used by recording action potentials in neurons in one location and subsequently triggering stimulation of neurons in another location [[94], [95], [96]]. This led to long-lasting synaptic strengthening between the targeted neurons [94], as well as between neurons and muscle cells [95], and between cortical motor neurons and spinal cord neurons [96]. These studies provide evidence for closed loop stimulation during free behaviour promoting plasticity of cortico-cortico, cortico-muscle or cortico-spinal connections. Artificial cortico-spinal connections could be driven by high gamma local field potential (LFP) activity recorded from motor or pre-motor cortex in monkeys [97], providing a practical method for such physiologically informed stimulation. A human pilot of this idea demonstrated long-term gains in spinal injury rehabilitation by delivering spinal stimulation linked to attempts at walking [98]. In simpler approaches that may be more easily implemented with current devices, plasticity can also be evoked using pulses delivered to two different brain regions with optimized timing. For instance, a combination of DBS and TMS pulses was used to alter cortico-subcortical motor circuit connectivity [99]. Pulse trains properly matched to circuit resonance have similar effects [53], and these approaches have at least shown safety in humans [52].

As with biomarkers, the study of timing-dependent neuromodulation and plasticity has recently emphasized oscillations. Stimulation appears to have dramatically different effects when delivered at different phases (points in the rise-fall cycle) of a neural oscillation. Triggering repetitive TMS pulses based on the ongoing individual alpha (10-13 Hz) phase was more able to modulate fronto-parietal oscillatory activity than non-phase-aware stimulation [100]. In the motor cortex, stimulation linked to the phase of a beta rhythm similarly induced intra-cortical changes [101]. For some disorders, these oscillations can even be read out peripherally. Cagnan et al. [102] demonstrated, for instance, that stimulation delivered at specific phases of limb tremor (which reflect oscillations in motor cortex) was more effective at cancelling that tremor.

Although phase-aware stimulation has not yet been extensively used with TMS, this is an excellent opportunity. TMS is readily combined with EEG, such that recorded brain oscillatory activity can be used to inform subsequent TMS stimulation. As TMS is already believed to work via neuroplastic effects [103], enhancing that neuroplasticity by phase-informed stimulation is an obvious next step. There is already at least one pilot clinical trial [104]. Perhaps the largest challenge is that phase-aware stimulation currently requires advanced signal processing equipment and can be very sensitive to investigator technique [100,105,106]. For this method to advance to clinical viability, neuromodulation device manufacturers will need to develop simple, turn-key methods for oscillation estimation. Open-source toolkits exist to facilitate that development [107]. Making real-time analysis of ongoing EEG signals available in the clinic should promote personalization of treatment across a wide spectrum of clinical applications.

So far in psychiatry, the majority of research conducted on neurophysiological biomarkers is focussed on depression as summarised in Table 1. Further studies are required to replicate and validate these research leads before they are ready for use in clinical practice. The evidence for biomarkers of other disease states, such as OCD, autism and ADHD, is currently sparce and needs to be explored further.

**Table 1:** Summary of research leads into potential neurophysiologic biomarkers in depression.

Diagnostic markers	Target engagement	Prognostics	Closed loop	Clinical Trial
Proximity of the individual alpha frequency (IAF) in the EEG to the 10 Hz rTMS frequency [41], replicated by [42] and 10 Hz sTMS frequency [45]	Suppression of an ERP related to aversive visual stimuli during epidural cortical stimulation (EpCS) [69]	Evoked or event-related potential in motor cortex: greater modulation of motor cortex excitability prior to rTMS predicted a better antidepressant response [60,61]	Stimulation at particular phase of the ongoing alpha oscillations [100]	Clinical Trial for closed loop stimulation is ongoing [104]

## 5 Limitations and opportunities

The success stories in adaptive systems to restore health are generally predicated on an understanding of physiology dynamics, from signals that correlate to symptoms to the response times of stimulation. The artificial pancreas in diabetes, cardiac pacemakers, and ventilators all demonstrate how this understanding, often with non-linear systems, can yield meaningful results. Results are more modest when the mapping is not understood, as exemplified by the current similarities between open loop and adaptive epilepsy systems. These successes also emerged as a series of refinements to technology, and the timelines for medical innovation require attention to achieving meaningful milestones to maintain interest and investment.

A key opportunity is to use the existing technology infrastructure as a platform to systematically explore physiological dynamics and refine therapies systematically. Therapy platforms provide access to the neural networks of interest, and data gathering and algorithmic prototyping that can leverage a digital infrastructure [108]. Another opportunity for platforms is that they allow for costs to be prorated among disease states, and for best practices to be shared among investigative teams. The NIH BRAIN and SPARC initiatives have helped develop and distribute platforms in partnership with industry.

Care must be taken when exploring sensing and adaptive systems, specifically designing for safe operating modes and limits to avoid over- or under-stimulation of the patients. Engineers have developed control frameworks for implementing these control limits, which are now being applied in research systems and shared as best practices [109]. For example, the ability to detect aberrant stimulation results, such as the onset of epileptiform after-discharges resulting from excessive stimulation and to turn down stimulation automatically, can help to ensure patient safety for novel therapy approaches [110].

## 6 Summary

The field of physiologically-informed neuromodulation is evolving rapidly and will both improve therapy efficacy and make patient management more efficient.

Patient stratification research remains important and will likely provide early value in enriching patient populations for clinical trials. For clinical decision making, such predictive biomarkers will have to be highly sensitive and selective. With the present modest sensitivity/specificity, withholding therapy from a patient based on a biomarker analysis is not clinically justifiable, particularly for non-invasive therapies such as TMS. For higher risk therapies, such predictions will be increasingly important as the patient and physician evaluate risks and benefits. The field of machine learning may lead to the identification of possibly very complex algorithms that have utility in personalizing treatments to patients. More high-quality multimodality datasets will be required to discover and develop new paradigms, and clinicians and researchers will need new skills to interpret the results of such research and avoid subtle methodological pitfalls [111].

Physiology-informed neuromodulation is already in use in clinical pilots, and more specific applications will follow to personalize treatment. Today, patient specific TMS treatment power is selected based on motor threshold measurements, often based on EMG. EEG-based and cardiac guided [112] treatment location and protocol selection is rapidly evolving, and may reach clinical use in the next few years. Phase-informed therapy holds promise to improve both invasive and non-invasive neuromodulation for several brain disorders. Phase-related synchrony metrics may also allow stimulation parameters to change depending on the state of the brain networks. Ultimately, these technologies may lead to real-time closed-loop interventions that can adjust neurostimulation to maintain these biomarkers within a target range.

Similarly, there is strong evidence that neuromodulation technologies change cortical excitability, and psychiatrists will likely benefit from the ability to evaluate treatment parameters or even combination therapies based on the patient's cortical excitability. Neurophysiological measurements will aid the psychiatrist in evaluating alternative treatment trials, as neurophysiological measures should be able to provide a quicker readout of therapy success.

Psychiatrists already have neurophysiological tools available to them such as EEG, evoked and event related potentials to assess a patient's brain function. Although none has yet reached a clinical level of evidence, this field is developing rapidly, and physicians will need to stay informed of these technological advances. Physiology-informed clinical practice may become viable in the next few years.

## Declaration of Competing Interest

Lothar Krinke is currently the CEO of Welcony Inc. Welcony Inc. is commercializing Electroencephalography, Transcranial Magnetic Stimulation and other Medical and Research Devices under the Magstim, EGI, Neurosign and Technomed brands and businesses. He also used to be an employee of Medtronic and holds Medtronic stock. Gaynor Foster and Alix Thomson are employed by Welcony Inc.

Alik Widge has multiple granted and pending patent applications in the use of physiologic markers to optimize neurostimulation, including for oscillation-related neurostimulation. None of these is licensed to any entity. He receives device donations from Medtronic, Inc., which previously employed Drs. Krinke and Denison.

Tim Denison has multiple granted and pending patent applications in the use of physiologic markers to optimize neurostimulation; the patents relevant for this work are licensed to Medtronic. He has consulted for Synchron, Cortec Neuro, Inspire, and Medtronic. He has research collaborations with and stock ownership in Bioinduction Ltd., research grants and collaborations with Magstim Ltd., and is a co-founder of Amber Therapeutics and Verity VR. These activities are managed with a conflict-of-interest plan at the University of Oxford. Tim Denison and Karen Wendt have received materials for research from Magstim Ltd.

Saydra Wilson has current grant funding to use physiologic markers to optimize neuromodulation supported by the 10.13039/100007249University of Minnesota's MnDRIVE (Minnesota's Discovery, Research and Innovation Economy) initiative.

Tim Denison, Karen Wendt, Alik Widge and Saydra Wilson received no direct financial support for the authorship of this article.

## Glossary

**Term**: **Definition**
Accelerometer: A device that measures acceleration. This can be used to measure posture and activity of a patient.
Closed-loop mode: As opposed to open-loop mode, the control of a system is regulated by feedback.
Control policy: Defines actions that can be taken to achieve a desired outcome.
Coordinated reset (CR): A model-based stimulation technique which specifically counteracts abnormal synchrony by desynchronization.
Deep brain stimulation (DBS): An invasive form of brain stimulation, where electrodes are surgically implanted into the brain.
Electroencephalography (EEG): Electrophysiological recording of brain activity by placing an array of electrodes on a patient's scalp.
Electromyography (EMG): Electrophysiological recording of electrical activity produced by skeletal muscles, measured by placing electrodes on the skin.
Endophenotypes: Or biotypes, a way of classifying patients according to physiology.
Event-related potential (ERP)/ Evoked potential: A stereotypical, reproducible electrical brain response to a specific stimulus.
Feedback and feedforward control: Pathways to adjust stimulation based on measured changes to/results of the system. Examples for feedback parameters are characteristics of brain signals, examples for feedforward parameters are circadian rhythms.
Individual alpha frequency (IAF): EEG measurement of the individual's dominant frequency in the alpha range.
Local field potential (LFP): Electrical activity recorded by small electrodes placed in extracellular space in brain tissue.
Motor evoked potentials (MEP): Electrophysiological recording of electrical activity produced by skeletal muscles following stimulation.
Open-loop mode: As opposed to closed-loop mode, the parameters of a system are static and not regulated by feedback.
Patient stratification: Prediction of which patients are likely responders to neuromodulation, or of which form of neuromodulation is right for a patient.
Phase-aware stimulation: Stimulation is applied at specific points in the rise-fall cycle of a biophysiological oscillation.
Stimulation target identification: Customization of a neuromodulation therapy target (anatomically or physiologically defined) to a patient's individual disease state or brain state.
Synchronized transcranial magnetic stimulation (sTMS): A non-depolarizing form of TMS, where the applied magnetic field changes sinusoidally.
Theta-burst stimulation (TBS): A TMS protocol where stimulation is applied in bursts of 3 pulses at 50 Hz, repeated every 200 ms.
Transcranial magnetic stimulation (TMS): A non-invasive form of stimulation where a magnetic pulse is applied to the target tissue through a coil placed over it. In repetitive TMS (rTMS), a train of pulses is applied which can be used to treat e.g. depression.
Vagal nerve stimulation (VNS): An implanted stimulator stimulates the vagus nerve with electrical impulses, used to treat e.g. epilepsy and depression.
